# Aspects of intradermal immunization with different adjuvants: The role of dendritic cells and Th1/Th2 response

**DOI:** 10.1371/journal.pone.0211896

**Published:** 2019-02-11

**Authors:** Zrinka Oreskovic, Katerina Nechvatalova, Josef Krejci, Vladimir Kummer, Martin Faldyna

**Affiliations:** 1 Department of Immunology, Veterinary Research Institute, Brno, Czech Republic; 2 Institute of Experimental Biology, Faculty of Science, Masaryk University, Brno, Czech Republic; Auburn University College of Veterinary Medicine, UNITED STATES

## Abstract

Intradermal (i.d.) application of vaccine is promising way how to induce specific immune response against particular pathogens. Adjuvants, substances added into vaccination dose with the aim to increase immunogenicity, play important role in activation of dendritic cells with subsequent activation of lymphocytes. They can, however, induce unwanted local reactions. The aim of the study was to determine the effect of i.d. administration of model antigen keyhole limped hemocyanine alone or with different adjuvants–aluminium hydroxide and oil-based adjuvants—on local histopathological reaction as well as dendritic cell activation at the site of administration and local cytokine and chemokine response. This was assessed at 4 and 24 hours after application. Selection of the adjuvants was based on the fact, that they differently enhance antibody or cell-mediated immunity. The results showed activation of dendritic cells and both Th1 and Th2 response stimulated by oil-based adjuvants. It was associated with higher expression of set of genes, incl. chemokine receptor CCR7 or Th1-associated chemokine CXCL10 and cytokine IFNγ. Application of the antigen with aluminium hydroxide induced higher expression of Th2-associated IL4 or IL13. On the other hand, both complete and incomplete Freund´s adjuvants provoked strong local reaction associated with influx of neutrophils. This was accompanied with high expression of proinflammatory IL1 or neutrophil chemoattractant CXCL8. Surprisingly, similarly strong local reaction was detected also after application of aluminium hydroxide-based adjuvant. The best balanced local reaction with sufficient activation of immune cells was detected after application of oil-based adjuvants Montanide and Emulsigen.

## Introduction

Skin is the largest organ covering an entire body. It provides the physical barrier between the body and its actually environment. Both skin layers, epidermis and dermis, are rich in several subpopulations of dendritic cells (DCs), which are professional antigen-presenting cells (APCs). They are specifically equipped to rapidly activate both innate and adaptive immune responses. This is achieved by releasing numerous chemokines and cytokines, and thereby recruitment of different cell types [1]. For instance, they are able to recruit neutrophils to the site of infection, tissue damage in skin at the injection site and are able to migrate and activate T helper cells (Th) towards a specific profile [2,3]. Porcine skin shares many anatomical characteristics of human skin such as structure and depth, together with cell populations such as Langerhans cells, dermal dendritic cells, macrophages, mast cells and skin-resident T cells [4–6]. Moreover, porcine dendritic cell subpopulations share similar properties to those of human dendritic cells. Consequently, the porcine model presents an efficient animal model for human immunological studies, especially in vaccine research [7–9]. Due to the skin properties described above, skin is the perfectly equipped habitat for antigen uptake and processing. It is also the ideal site for vaccine delivery. Despite having many advantages over other methods of vaccine delivery, intradermal immunization (i.d.) is still seldom-used.

Also, with the same amount of antigen, it is possible to prepare more i.d. doses than intramuscular (i.m.) ones. This has a dose-sparing effect, while still eliciting efficient, and in cases of influenza vaccine for instance, a better immune response than by the i.m. route [10–15]. To increase vaccine efficiency, the presence of an adjuvant is required in every vaccine regardless of the administration route. Also, adjuvants modulate the immune response by skewing it towards a specific cellular profile. For example, aluminium salts that are commonly used in human vaccines primarily elicit the Th2 type of response, while other formulations such as saponins or different oil-based emulsions are shown to elicit both Th1 and Th2 type of response [16,17]. However, there is an increasing demand for new target-specific formulations able to elicit particular cellular types, e.g. CD8+ cells, Th1, Th2 and Th17 helper profile, as well as vaccines specifically targeting DCs, thus contributing to novel vaccine development, such as cancer vaccine [18,19]. Since skin is rich in different subpopulations of dendritic cells, which are pivotal activators of naïve T-lymphocytes towards different effector subsets, we examined the changes *in situ* after i.d. administration of different oil-based adjuvants and Al(OH)_3_ affecting the dendritic cell maturation and activation, as well as potential modulation of immune response towards Th1 and Th2 response orchestrated by skin DCs.

Experiments previously performed in our laboratory demonstrated that oil-based adjuvants delivered intradermally increased both humoral and cellular immune responses accompanied by the production of primary antibody IgG1 and IgG2 antibody confirming simultaneous activation of both Th1 and Th2 responses which did not differ in strength in comparison to intramuscular delivery [20,21]. On the other hand, after application of some of them, strong local reactions were detected.

Therefore, to gain a new insight into the activation of the immune response after intradermal vaccine delivery, model antigen KLH was combined with different adjuvants and administered *in vivo*. Results were obtained by histopathological assessment of local reactions in the skin in combination with relative quantification of mRNA expression for different chemokine and cytokine associated with local inflammatory reaction and activation of dendritic cells and T cell at the injection site.

## Materials and methods

### Animals and experimental design

Six healthy genetically non-related, six-week-old Large White domestic pigs were used to examine the effects of i.d. immunization with different adjuvants. Animals originated from a farm with a good current epidemiological situation and were housed under controlled conditions in the accredited experimental animal facility of the Veterinary Research Institute, Brno, Czech Republic. The pigs were allowed to acclimatize in the animal facilities for two weeks prior to the experiment. Animals were challenged intradermally with model antigen KLH, (Pierce, France), alone or combined with different types of adjuvants. The following adjuvants were used: (1) complete and (2) incomplete Freund’s adjuvant (CFA and IFA) (Sigma–Aldrich, USA), (3) aluminium hydroxide (Alhydrogel, Denmark, hereinafter referred to as Al(OH)_3_), (4) Montanide ISA 206 (Seppic, France, hereinafter referred to as ISA) and (5) Emulsigen (MVP Laboratories, USA, hereinafter referred to as Emuls.). KLH was injected at the concentration of 0.04 mg and the total volume of one intradermal immunization dose was 0.15 mL. Al(OH)_3_ and Emulsigen adjuvants were mixed with antigen at 1:3 ratio and other adjuvants 1:1. The i.d. injections were administered into the prescapular region, using short intradermal needles. Points of the application were marked with permanent color mark. Skin samples for histopathology and quantitative RT-PCR were collected 4h and 24h post-immunization. Samples were taken directly from the points of application using the 5 mm bioptic needle. The skin biopsies were performed under sedation of animals with combination of telazol-ketamin-xylazin. The experiment was performed in compliance with the Act No. 246/1992 Coll. of the Czech National Council on the protection of animals against cruelty, and with the agreement of the Branch Commission for Animal Welfare of the Ministry of Agriculture of the Czech Republic (approval no. MZe-822). Commercial processing of the pigs after the experiment was finished was in compliance with national legislation on animal experimentation.

### Histopathology and local skin reactions

**Fig 1:**
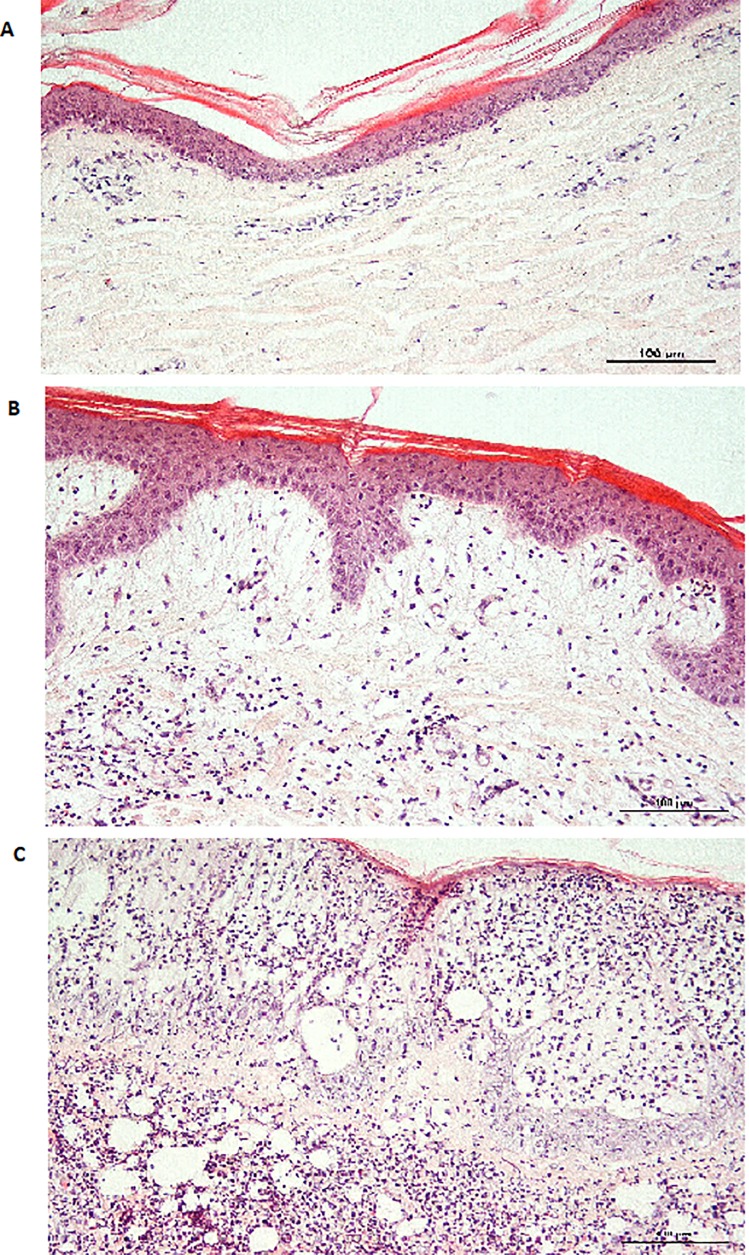
Examples of histopathological lesions at the site of intradermal administration. Examples of semi-quantification of a cellular influx at the site of intradermal administration of KLH alone or in combination with different adjuvants. Influx intensity was established as low (A), mild/moderate (B) or massive (C).

All samples were examined for gross and microscopic lesions. Skin biopsies were fixed in 10% neutral buffered formalin for 24 h and embedded in paraffin wax. Sections (5 μm) were cut, stained with hematoxylin and eosin and toluidine blue, and examined by light microscopy using a microscope Olympus IX51. The intensity of cellular influx was semi-quantified as follows: (+) low influx; (++) mild/moderate influx; (+++) massive influx. Examples are shown in Fig 1.

### RNA isolation and quantitative Real-time PCR

To determine the local proinflammatory response, activation of dendritic cells and Th1/Th2 response, specific cytokine and chemokine production was quantified by real-time RT-PCR. Prior to RNA isolation, skin samples were homogenised in TRI-Reaget (Sigma- Aldrich). RNA was isolated from skin samples using the RNeasy Mini Kit (Qiagen) according to the manufacturer’s recommendation and then transcribed into cDNA using M-MLV reverse transcriptase (Invitrogen) and oligo-dT primers (GeneriBiotech). To determine specific cytokine production, Quantitative Real-time PCR (RT-PCR) was performed using the QuantiTect SYBR Green PCR Kit (Qiagen) and gene-specific primers (Generi Biotech) (Table 1) on a LightCycler 480 II in a 384-well plate block (Roche). The expression of each cytokine was calculated relative to the reference gene Hypoxanthine phosphoribosyltransferase (HPRT) presented as 2^(-ΔCt)^. HPRT was selected as a reference gene based on evaluation by the RefFinder tool (http://www.leonxie.com/referencegene.php) prior to the measurement. The other tested genes TATA-binding protein 1 (TBP), hydroxymethylbilane synthase (HMBS) and beta-actin (ACTB) showed less stabile transcription. Primers were designed either *de novo* using the NCBI primer designing tool (http://www.ncbi.nlm.nih.gov/tools/primer-blast/) or adopted from our previous experiments [22,23].The threshold cycle values (Ct) of the genes of interest were first normalized to the Ct value of HPRT reference mRNA (ΔCt), and the normalized mRNA levels were calculated as 2(^−ΔCt^). The results are presented as mean values of fold increase of the gene of interest.

**Table 1:** List of primers used in the study.

HPRT	Forward	CGGCTCCGTTATGGCG
	Reverse	GGTCATAACCTGGTTCGTCATCA
IL1α	Forward	TGTGAAGTGTTGACAGGCCGTATGTACC
	Reverse	CTCAGCACATGCTCAGCGAGTGAC
IL4	Forward	TCGGCACATCTACAGACACC
	Reverse	CTTCTTGGCTTCATGCACAG
IL13	Forward	ACCAGCATGCAGTACTGTGCCGC
	Reverse	ACTTGCTCGCTTGGGGGCTTGTG
IL18	Forward	ATGCCTGATTCTGACTGTTC
	Reverse	CTGCACAGAGATGGTTACTGC
IFNγ	Forward	CCATTCAAAGGAGCATGGAT
	Reverse	GAGTTCACTGATGGCTTTGC
TNFα	Forward	CCCCCAGAAGGAAGAGTTTC
	Reverse	CGGGCTTATCTGAGGTTTGA
CCL3 (MIP1α)	Forward	TTTTGAGACCAGCAGCCAGT
	Reverse	TCAGCTCCAGGTCAGAGATG
CCL5 (RANTES)	Forward	ACCACACCCTGCTGTTTTTC
	Reverse	GGCGGTTCTTTCTGGTGATA
CCL17 like	Forward	CTCCTCCTGGGGGCTTCCCTGC
	Reverse	CAGCACTCCCGGCCCACGTTG
CXCL8 (IL8)	Forward	ATGCCTGATTCTGACTGTTC
	Reverse	CTGCACAGAGATGGTTACTGC
CXCL9	Forward	AGCAGTGTTGCCTTGCTTTTGGGTATCATC
	Reverse	GCTGGTGTTGATGCAGGAACAACGTCC
CXCL10	Forward	CCCACATGTTGAGATCATTGC
	Reverse	CATCCTTATCAGTAGTGCCG
CXCL16	Forward	TCGCGGAGAATGTGGACGTGCTC
	Reverse	TCGTCTGGGCAGGGGTGCTACTG
CD80	Forward	AATGGTCAAAGCTGACTTTCCTG
	Reverse	GGTTGAGCACCTTATCCTTTTGA
CD86	Forward	CCCCTCTAATGAATGTGGTGAAAC
	Reverse	GATCGTTCATGGACTTCTGCTCT
CCR7	Forward	GTGGTGGCTCTCCTTGTCAT
	Reverse	GAAGCACACGGACTCGTACA
NFκBi	Forward	ACGAGCAGTGGTGAAGGAG
	Reverse	TCATGGATGATGGCCAAGT

### Statistical analysis

All calculations were performed with Prism (Graph Pad Software, Inc.) software. Results of cytokine and chemokine expressions are presented as quantification values obtained by qRT-PCR for each sample and were evaluated by the nonparametric Kruskal–Wallis test. Differences between the particular groups were calculated. The differences with p < 0.05 were considered statistically significant.

## Results

### Cellular influx into the injection site

Following the intradermal administration of KLH alone after 4 hours, only weak, light and diffuse infiltration of neutrophils was detected. Administration of KLH combined with CFA provoked a median skin reaction, with neutrophil infiltrate already visible within 4 hours post-injection. KLH combined with IFA caused reactions similar to those of CFA, with moderate infiltration of neutrophils 4 hours after administration. Only a slight infiltration of neutrophils was visible 4h after i.d. administration of KLH with aluminum. Twenty-four hours after the administration, however, strong influx of neutrophils and necrosis of the ligament with the tendency of abscess formation was observed. On the other hand, KLH combined with Emulsigen led to a balanced neutrophil response, with the mild influx of leucocytes, eosinophils and monocytes. Similarly to Emulsigen, KLH combined with Montanide ISA caused mild cellular influx within the first 4 hours (Table 2).

**Table 2:** Semi-quantification of cellular influx at the site of administration of KLH alone or in combination with different adjuvants 4 and 24 hours after application.

	Intact skin	KLH	KLH+CFA	KLH+IFA	KLH+Al(OH)_3_	KLH+Emuls.	KLH+ISA
4 hours	-	+	+++	+++	++	++	++
24 hours	-	++	+++	+++	+++	++	++

Intensity of cellular influx after application of complete and incomplete Freund’s adjuvant (CFA and IFA, respectively) aluminium hydroxide (Al(OH)_3_), Emulsigen (Emuls.) and Montanide ISA 206 (ISA). The influx was semi-quantified as follows: (+) low influx; (++) mild/moderate influx; (+++) massive influx as shown in Fig 1.

### Local reactions in the skin

#### Local cytokine and chemokine response 4 hours post-immunization

**Fig 2:**
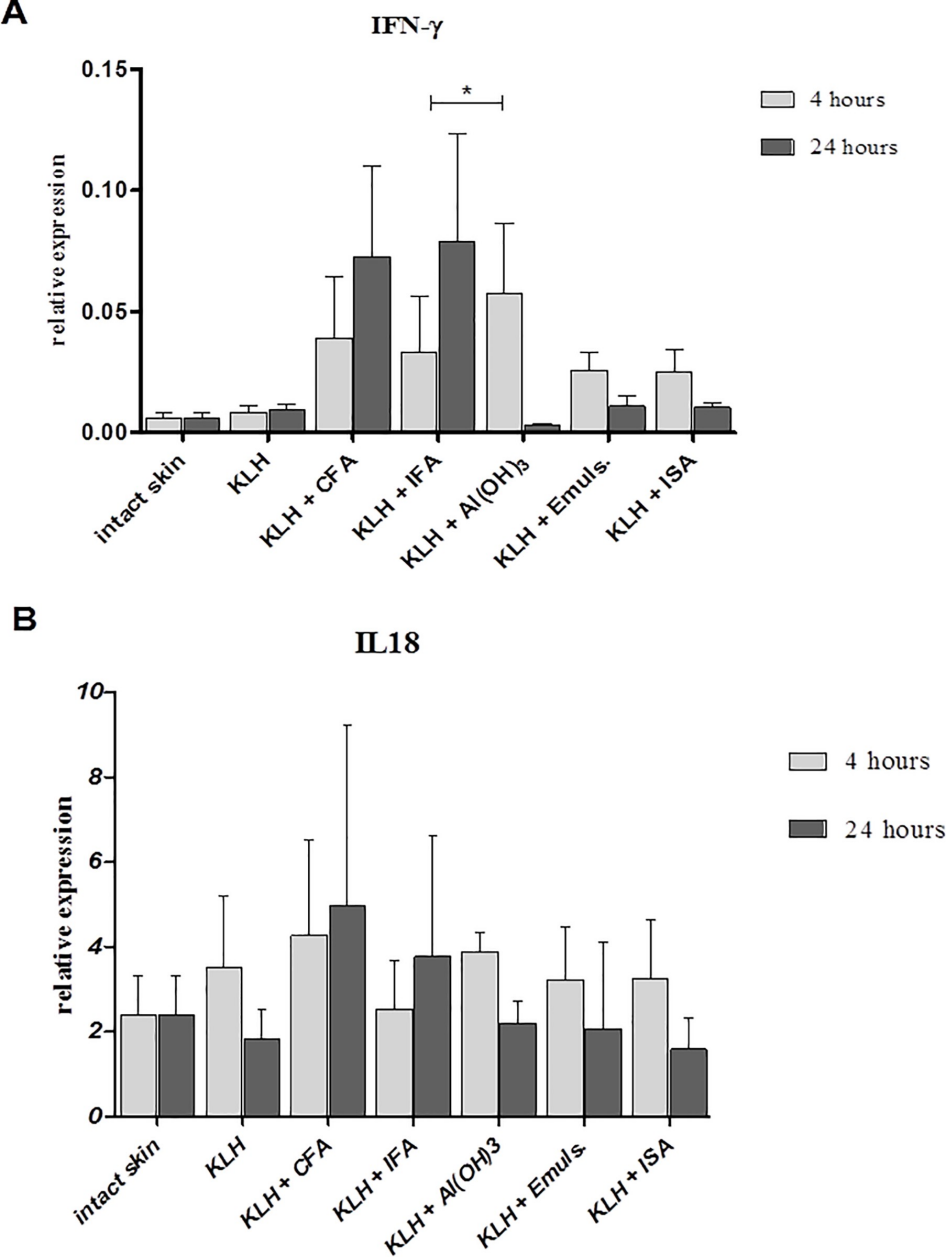
Relative expression of IFNγ and IL18. Relative expression of IFNγ (A) and IL18 (B) at the site of intradermal administration of KLH alone or in combination with different adjuvants 4 and 24 hours after application. Results of quantitative real-time PCR are presented as mean ± SD values of fold increase of the gene of interest against the housekeeping gene (n = 6 per group). Statistically significant differences between the groups are marked with asterisks (p < 0.05 in Kruskal-Wallis test).

**Fig 3:**
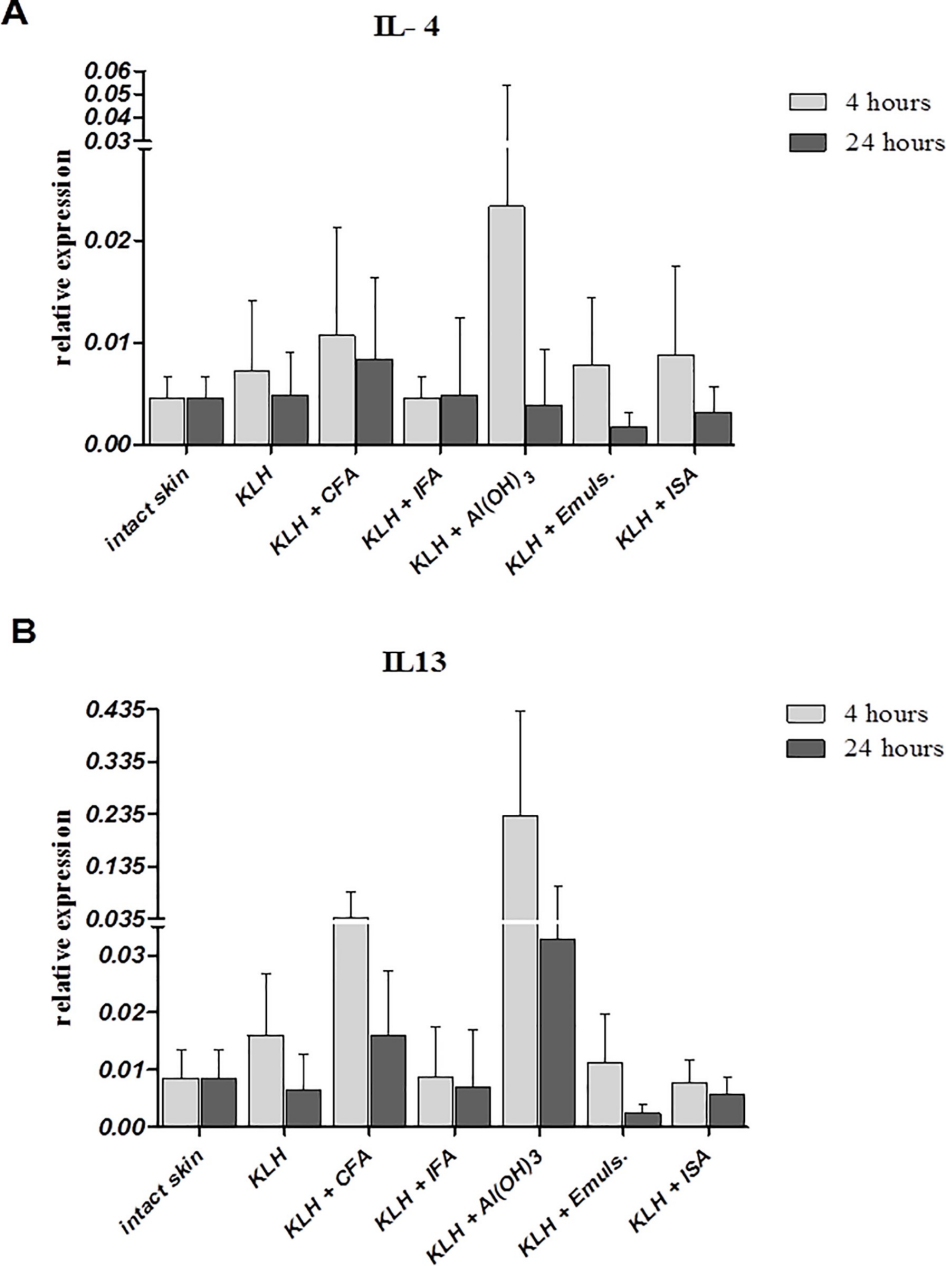
Relative expression of IL4 and IL13. Relative expression of IL4 (A) and IL13 (B) at the site of intradermal injection of KLH alone or in combination with different adjuvants 4 and 24 hours after application. Results of quantitative real-time PCR are presented as mean ± SD values of fold increase of the gene of interest against the housekeeping gene (n = 6 per group).

All primary data are shown in S1 Table. The proinflammatory response at the site of injection was detected by local cytokine (IL1α, IL4, IL13, IL18, IFNγ and TNFα) and chemokine (CCL3, CCL5, CXCL8 and CXCL16) production within 4 and 24 hours. The increase of IL18 and IFNγ (Fig 2) was detected after the administration of all adjuvants within the first 4 hours and the overall expression was higher than both IL13 and IL4 if expressed to HPRT. As expected, both Al(OH)_3_ and Freund’s complete adjuvant induced higher expression of IL4 and IL13 (Fig 3) than the other adjuvants. Interestingly, Al(OH)_3_ also induced higher expression IFNγ than oil-based adjuvants within the first 4 hours. Other cytokines, IL1α and TNFα, induced similar expression with increased production induced by all adjuvants within 4 hours. Similarly to IFNγ and IL4, expression of both IL1α and TNFα induced by Al(OH)_3_ after 4 hours was higher than expression induced by oil-based adjuvants (Table 3). CXCL8 and CXCL16 chemokine expression was similar to proinflammatory cytokine production. Both chemokines were more strongly induced by Al(OH)_3_ within the first four hours than by Emulsigen and Montanide ISA. However, CXCL8 was also induced by both Freund’s adjuvants, with CFA provoking the highest expression, while CXCL16 was more prominent after the administration of Emulsigen and Montanide than Freund’s adjuvants (Table 3).The expression of proinflammatory chemokines CCL3 and CCL5 was similar to proinflammatory cytokine expression, with all adjuvants elevating the expression of both chemokines 4 hours post-immunization, with Emulsigen and Montanide provoking higher expression than the remaining adjuvants (Table 3).

**Table 3:** The expression of selected genes after 4 hours at the site of intradermal application of KLH alone or in combination with different adjuvants.

4 hours		Intact skin	KLH	KLH+CFA	KLH+IFA	KLH+Al(OH)3	KLH+Emuls	KLH+ISA
IL1α	mean	0.349	0.389^a^**	0.693	0.484	1.588^a^	0.603	0.599
	SD	0.07	0.18	0.27	0.09	0.42	0.13	0.25
TNFα	mean	0.018	0.039	0.055	0.053	0.110	0.071	0.084
	SD	0.01	0.02	0.01	0.04	0.12	0.06	0.04
CXCL8	mean	0.017	0.199^a^*	5.396^a^	1.700	2.718	0.400	0.973
	SD	0.02	0.37	4.19	1.93	3.34	0.43	1.02
CXCL16	mean	1.528	1.251	1.377	1.245	4.847	2.188	2.353
	SD	0.54	0.37	0.76	0.57	1.95	1.02	0.49
CCL3	mean	0.030	0.227	0.604	0.683	1.156	1.672	1.928
	SD	0.02	0.15	0.42	0.57	1.27	1.14	1.14
CCL5	mean	0.331	0.483	0.356^a^*	0.353^b^*	0.836	0.979	1.150^ab^
	SD	0.28	0.33	0.25	0.33	0.44	0.21	0.16

Results of quantitative real-time PCR are presented as mean ± SD values of fold increase of the gene of interest against the housekeeping gene (n = 6 per group). Statistically significant differences between the groups are marked as letters with asterisks (* p < 0.05 and ** p < 0.01 in Kruskal-Wallis test).

#### Local cytokine and chemokine response 24 hours-post immunization

After 24 hours, the overall proinflammatory response was differently expressed in comparison to the first 4 hours. Expression of IL18 and IFNγ induced by both Freund’s adjuvants was higher than expression induced by all other adjuvants. Moreover, the expression of both cytokines was increased after 24 hours post-immunization with Freund’s adjuvants. The decrease and generally lower expression of both cytokines were observed after i.d. administration of aluminum in comparison to 4 hours. Additionally, levels of IFNγ provoked by Emulsigen and Montanide ISA were slightly higher than levels provoked by Al(OH)_3_, suggesting Th1-inducing properties of both Emulsigen and Montanide ISA (Fig 2). Furthermore, all adjuvants seemed to decrease the overall expression of both IL4 and IL13 after 24 hours in comparison to 4 hours. However, CFA and Al(OH)_3_ induced higher expression of both cytokines than the other adjuvants, thus following the expression observed after 4 hours (Fig 3).The expression of IL-1α and TNFα was increased by CFA and IFA after 24 hours in comparison to 4 hours, while after i.d. administration of other adjuvants, it was lower or similar to 4 hours (Table 4). CXCL8 and CXCL16 chemokine expression was highly induced 24 hours post-immunization by CFA and IFA. Other adjuvants elevated the level of CXCL8 after 24 hours, but CXCL16 was decreased after 24 hours (Table 4).

**Table 4:** The expression of selected genes after 24 hours at the site of intradermal application of KLH alone or in combination with different adjuvants.

24 hours		Intact skin	KLH	KLH+CFA	KLH+IFA	KLH+Al(OH)3	KLH+Emuls	KLH+ISA
IL1α	mean	0.349	0.303	4.645^a^*	3.565	0.232^a^	0.523	0.334
	SD	0.07	0.10	5.52	3.96	0.16	0.34	0.35
TNFα	mean	0.018	0.018^a^**	0.213^a^	0.063	0.007	0.018	0.029
	SD	0.01	0.01	0.20	0.06	0.00	0.01	0.03
CXCL8	mean	0.017	0.555	34.744	23.075	5.936	1.640	3.200
	SD	0.02	0.51	15.77	26.42	11.17	1.24	1.94
CXCL16	mean	1.528	1.916^a^*	6.162	2.237	1.392	1.759	1.255^a^
	SD	0.54	0.57	3.43	1.05	0.72	0.59	0.47
CCL3	mean	0.030	0.248^ab^	19.991^a^**	10.943^b^*	0.726	1.588	1.950
	SD	0.02	0.23	20.07	14.69	0.61	1.75	1.57
CCL5	mean	0.331	0.816^a^	13.870a*	4.907	1.649	2.254	2.142
	SD	0.28	0.77	2.56	3.07	1.34	2.85	0.60

Results of quantitative real-time PCR are presented as mean ± SD values of fold increase of the gene of interest against the housekeeping gene (n = 6 per group). Statistically significant differences between the groups are marked as letters with asterisks (* p < 0.05 and ** p < 0.01 in Kruskal-Wallis test).

Proinflammatory chemokines CCL3 and CCL5 were increased after 24 hours by both CFA and IFA, but the expression of CCL3 was either slightly lower (Al(OH)_3_) or comparable (Emulsigen and Montanide) in comparison to 4 hours, while CCL5 was increased after i.d. administration of any of the adjuvants (Table 4).

**Fig 4:**
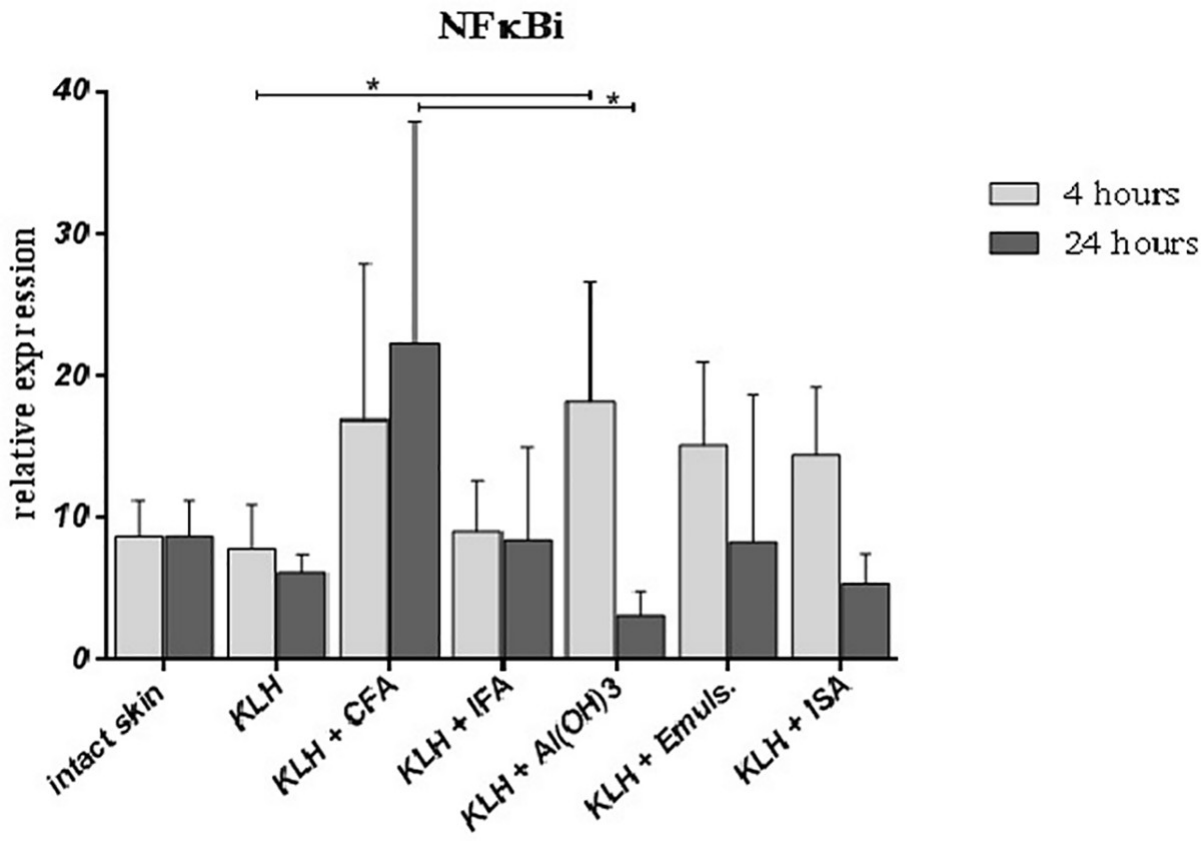
Relative expression of NFκBi. Relative expression of NFκBi at the site of intradermal administration of KLH alone or in combination with different adjuvants 4 and 24 hours after application. Results of quantitative real-time PCR are presented as mean ± SD values of fold increase of the gene of interest against the housekeeping gene (n = 6 per group). Statistically significant differences between the groups are marked with asterisks (p < 0.05 in Kruskal-Wallis test).

In order to further examine the *in situ* inflammatory response, the transcription factor nuclear factor-kappa B inhibitor was measured. The decrease in the expression of NFκBi was detected 24 hours post-immunization using both Emulsigen and Montainde ISA as well aluminum in comparison to the first 4 hours, but CFA and IFA induced higher expression similar to that observed after 4 hours (Fig 4), thus correlating with the observed proinflammatory response.

### Dendritic cell activation

**Fig 5:**
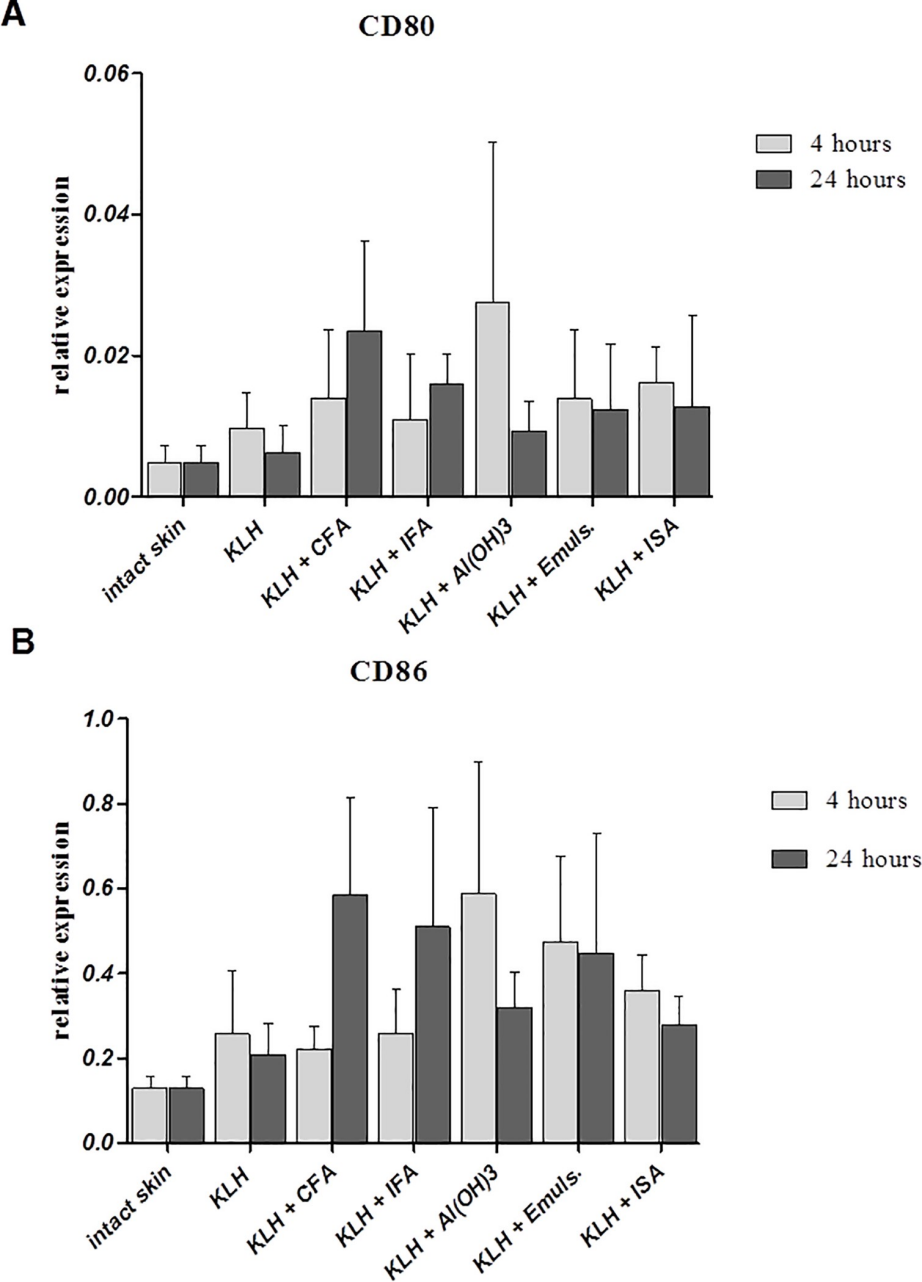
Relative expression of CD80 and CD86. Relative expression of CD80 (A) and CD86 (B) at the site of intradermal administration of KLH alone or in combination with different adjuvants 4 and 24 hours after application. Results of quantitative real-time PCR are presented as mean ± SD values of fold increase of the gene of interest against the housekeeping gene (n = 6 per group).

**Fig 6:**
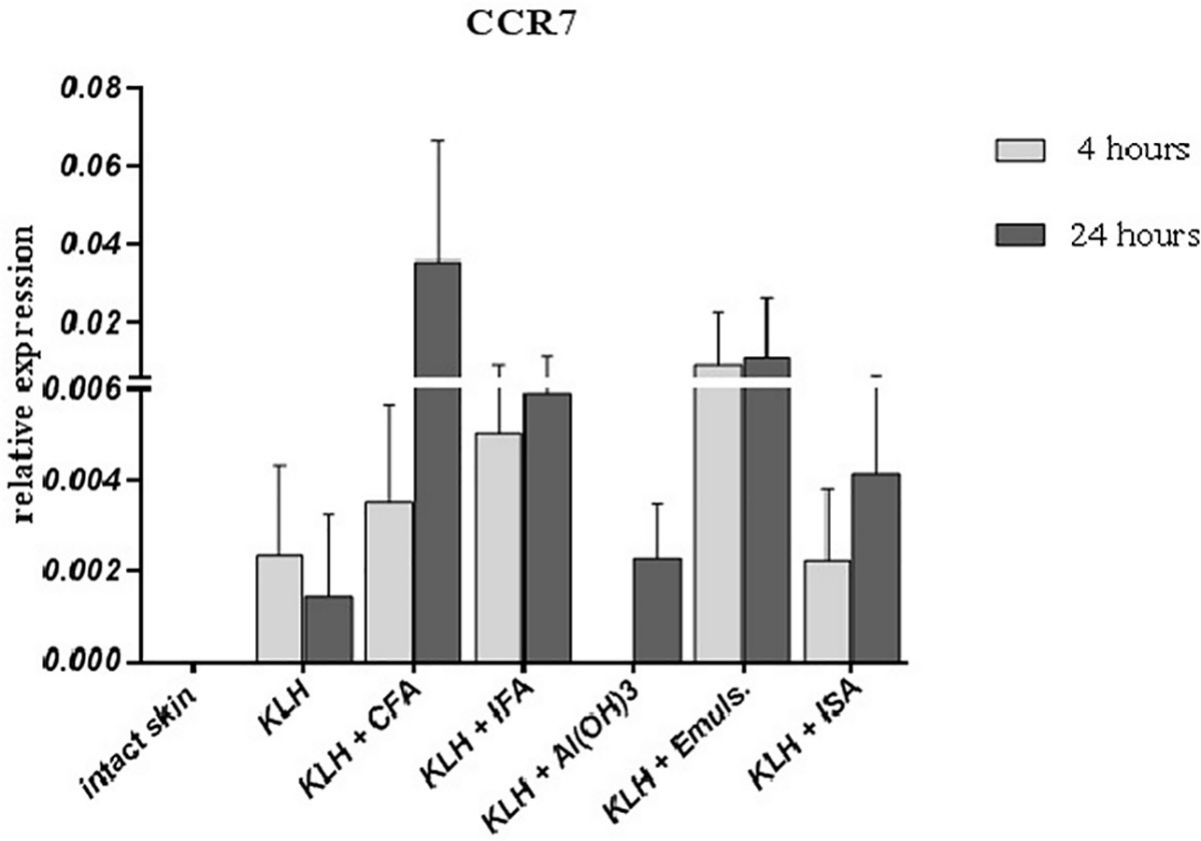
Relative expression of CCR7. Relative expression of CCR7 in the site of intradermal administration of KLH alone or in combination with different adjuvants 4 and 24 hours after application. Results of quantitative real-time PCR are presented as mean ± SD values of fold increase of the gene of interest against housekeeping gene (n = 6 per group).

Dendritic cells are the most abundant in both epidermis and dermis and therefore the i.d. route of vaccine delivery specifically targets skin-resident DCs. Activation of dendritic cells was determined by expression of co-stimulatory molecules CD80 and CD86, as well as CCR7, a receptor expressed by activated DCs. Within the first 4 hours, all adjuvants upregulated the expression of CD80/86, with Al(OH)_3_ being the most prominent (Fig 5). Interestingly, dendritic cell activation marker, CCR7, was not detected after 4 hours post-immunization with Al(OH)_3_. However, it was upregulated by some oil-based adjuvants (Fig 6).

Similarly to proinflammatory cytokines described above, both CD80 and CD86 were upregulated by Freund’s adjuvants after 24 hours, but expression after i.d administration of other adjuvants was either downregulated (Al(OH)_3_) or remained similar (Emulsigen and Montanide ISA) when compared to the situation 4 hours post-immunization. CCR7 was upregulated by Freund’s adjuvants and Montanide ISA after 24 hours, while Emulsigen did not induce any changes in expression during 4 and 24 hours. Al(OH)_3_, however, induced expression of CCR7 24 hours after delivery to levels similar to that induced by Montanide during the first 4 hours.

### T cell activation

DCs are pivotal in T lymphocyte activation and proliferation towards specific effector profile. Since rapid activation of DCs and specific IFNγ/IL4 production at the site of injection were observed, both Th1 and Th2 inducing chemokines were analyzed to determine possible T-cell profile induced by activated DCs.

Expression of Th1 chemokines CXCL9 and CXCL10 correlated with the expression of the proinflammatory cytokines and costimulatory molecules CD80/86 and it was induced by all oil-based adjuvants after 4h hours (Table 5). Emulsigen and ISA induced higher expression than other adjuvants. After 24 hours, the expression of all chemokines induced by Al(OH)_3_, Emulsigen and Montanide ISA was lower than after the first 4 hours. Both Freund’s adjuvants induced higher responses 24 hours post-immunization, which also correlates with the observed expression of proinflammatory cytokines (Table 6).

**Table 5:** The expression of CXCL9 and CXCL10 after 4 hours at the site of intradermal administration of KLH alone or in combination with different adjuvants.

4 hours		Intact skin	KLH	KLH+CFA	KLH+IFA	KLH+Al(OH)3	KLH+Emuls	KLH+ISA
CXCL9	mean	0.174	0.193	0.217	0.212	0.206	0.559	0.378
	SD	0.07	0.11	0.14	0.14	0.14	0.20	0.23
CXCL10	mean	0.714	1.416	1.968	2.896	1.176	2.623	3.127
	SD	0.43	0.53	1.91	2.89	0.41	1.04	2.23

Results of quantitative real-time PCR are presented as mean ± SD values of fold increase of the gene of interest against the housekeeping gene (n = 6 per group).

**Table 6:** The expression of CXCL9 and CXCL10 after 24 hours at the site of intradermal administration of KLH alone or in combination with different adjuvants.

24 hours		Intact skin	KLH	KLH+CFA	KLH+IFA	KLH+Al(OH)3	KLH+Emuls	KLH+ISA
CXCL9	mean	0.174	0.198	0.345	0.227	0.037	0.100	0.194
	SD	0.07	0.09	0.32	0.26	0.03	0.14	0.24
CXCL10	mean	0.714	1.939	9.514^a^***	5.136^b^**	0.351^ab^	1.760	2.545
	SD	0.43	1.73	8.88	4.61	0.19	1.17	1.40

Results of quantitative real-time PCR are presented as mean ± SD values of fold increase of the gene of interest against the housekeeping gene (n = 6 per group). Statistically significant differences between the groups are marked as letters with asterisks (** p < 0.01 and *** p < 0.001 in Kruskal-Wallis test).

Th2 inducing chemokines CCL17 and CCL22 were, however, differently expressed. CCL17 was upregulated by aluminum and Emulsigen 4h post immunization when compared to other adjuvants, while CCL22 was highly induced by both Freund’s adjuvants (Table 7). 24 hours post-immunization, levels of CCL17 were induced by both Freund’s adjuvants but decreased after Al(OH)_3_. Emulsigen and Montanide ISA induced a similar expression (Emulsigen) or a slightly higher expression (Montanide ISA) (Table 8).

**Table 7:** The expression of CCL17 and CCL22 after 4 hours at the site of intradermal administration of KLH alone or in combination with different adjuvants.

4 hours		Intact skin	KLH	KLH+CFA	KLH+IFA	KLH+Al(OH)3	KLH+Emuls	KLH+ISA
CCL17	mean	0.013	0.080	0.043	0.081	0.351	0.255	0.085
	SD	0.01	0.08	0.04	0.07	0.32	0.32	0.08
CCL22	mean	0.017	0.030	0.265	0.205	0.065	0.123	0.059
	SD	0.01	0.03	0.25	0.28	0.04	0.15	0.05

Results of quantitative real-time PCR are presented as mean ± SD values of fold increase of the gene of interest against the housekeeping gene (n = 6 per group).

**Table 8:** The expression of CCL17 and CCL22 after 24 hours at the site of intradermal administration of KLH alone or in combination with different adjuvants.

24 hours		Intact skin	KLH	KLH+CFA	KLH+IFA	KLH+Al(OH)3	KLH+Emuls	KLH+ISA
CCL17	mean	0.013	0.052ab	0.541a*	0.398a*	0.125	0.190	0.259
	SD	0.01	0.03	0.59	0.14	0.07	0.15	0.23
CCL22	mean	0.017	0.028	0.180	0.079	0.033	0.083	0.050
	SD	0.01	0.03	0.12	0.04	0.03	0.13	0.03

Results of quantitative real-time PCR are presented the as mean ± SD values of fold increase of the gene of interest against housekeeping gene (n = 6 per group). Statistically significant differences between the groups are marked as letters with asterisks (* p < 0.05 in Kruskal-Wallis test).

## Discussion

Adjuvants are commonly used to enhance vaccine efficacy and to promote and modulate the immune response, but evidence of their effect on the immune response and modulation of a specific T-cell profile via intradermal vaccination is still scarce. The administration route of the vaccine is important in generating a proper immune response. For example, Th1 response based on IgG2 levels is dependent on the delivery route [24]. The results presented here correlate with our previous findings, showing a differential *in situ* response induced by respective adjuvants. Both of Freund’s adjuvants are known to cause a strong local reaction and are not recommended for human vaccine formulations [25,26]. Also in our study, infiltration of neutrophils observed after 4 hours declined after 24 hours after administration of KLH only. On the other hand, KLH combined with CFA provoked strong local reaction associated with cellular influx with predominance of neutrophils after 24 hours. IFA caused reactions similar to those of CFA administration, also leading to massive infiltration of neutrophils into the site of injection within 24 hours. Interestingly, 24 hours after the administration, large condensation of neutrophils was detected in dermis also in the case of Al(OH)_3_. Furthermore, deposits with signs of neutrophil decay, necrosis of the ligament and the tendency of abscess formation were observed. Al(OH)_3_ is a commonly used adjuvant known for its mild reaction and Th2 type of response [27]. Based on our results, we might even suggest that Al(OH)_3_ is potentially not a suitable adjuvant for i.d. vaccination, regardless of its common use in i.m. vaccine formulations. However, this is only a suggestion and should be further examined. Contrary to the other adjuvants, the cellular influx and local reaction observed 4 hours after the administration of Emulsigen and Montanide was adequately enhanced without any evidence of a local hyperactivity at the site of injection. Lymphocyte, monocyte and eosinophil influx increased within 24 hours too. Based on the results we suggest that both Emulsigen and Montanide ISA could be used as potential adjuvants for the i.d. delivery route in both human and veterinary vaccine formulations.

Intensity of the cellular influx into the injection site observed by histopathology corresponded to intensity of cytokine release detected by qRT-PCR. Generally, proinflammatory response represented by IL1α, CXCL8 or CXCL16 production within 4 and 24 hours was the strongest after application of Freund´s adjuvants and/or Al(OH)_3_.

The acting mechanisms and potential time–dependency of different adjuvants in the activation of the immune response are visible in their ability to induce maturation and activation of skin-resident dendritic cells. DCs respond to initial proinflammatory signals produced by other cells such as keratinocytes, which are the source of TNFα, and skin-resident macrophages releasing CCL3, CCL5 and CXCL8, as well as to antigen itself upon uptake [28,29]. Both keratinocytes and mature dendritic cells produce IL-1α in the skin providing a solid proinflammatory response [30,31]. Also, immature DCs produce CXCL8 to promote neutrophil migration into the site of infection or vaccination [8]. Furthermore, keratinocytes upregulate the levels of CXCL16 as a response to induced levels of TNFα and IFNγ, and thus additionally they upregulate neutrophil recruitment into the skin [32]. Moreover, CXCL16 is also produced by mature dendritic cells attracting CXCR6+ T-cells, thus contributing to their activation and retention at the site of inflammation [33,34].

Based on results presented, it seems that i.d. immunization combined with Emulsigen or Montanide ISA provides a strong proinflammatory response, with the rapid secretion of different chemokines activating both the innate immune response and neutrophil recruitment into the site of administration. This is associated with activation of DCs. Additionally, 24 hours post-immunization, Emulsigen and Montanide ISA provoked a stronger reaction than Al(OH)_3_, but without severe local reaction, suggesting their ability to provide sufficient immune response without detrimental local reaction at the site of administration. Furthermore, all adjuvants upregulated the costimulatory molecules CD80 and CD86, but oil-based adjuvants activated skin-resident DCs faster, i.e. within the first four hours post-immunization, which was demonstrated by the expression of CCR7. As skin DCs mature following the antigen uptake and both chemokine and cytokine stimulation, they are exposed to in their microenvironment, and upregulate costimulatory molecules CD80 and CD86, but only upon activation they start to express CCR7, the receptor crucial for migration of DCs towards secondary lymphoid tissues [35–37]. It is clear that oil-based adjuvants provoke rapid DC maturation and activation. The observed decrease in the expression of chemokines and cytokines could be related to brisk activation of dendritic cells and early expression of CCR7 leading to their migration towards draining lymph nodes. Therefore, it is possible that a certain part of DC population could be involved in swift migration towards secondary lymphoid tissues thus inducing a relatively rapid subsequent activation of naïve T-cells towards both profiles. This is further supported by the decline in the expression of NFκB inhibitor observed after 24h, as chemokine production by DCs and their maturation are dependent on the NFκB pathway [38,39]. Moreover, mild local reaction and the decline of the proinflammatory response at the site of Emulsigen and Montanide ISA administration compared to Freund’s adjuvants suggests that the mechanism of immune response activation could be time-dependent and in fact reaching its peak within only a few first hours after the administration. The inflammatory response provoked *in situ* is relatively short in time, but sufficient enough to activate skin-resident DCs and subsequently activate both humoral and cellular responses towards both Th1 and Th2 responses.

Previous studies have shown that DCs activate Th1 or Th2 lymphocytes depending on chemokine profile. Th1-activating DCs release chemokines such as CXCL9 and CXCL10 [40–42] or CCL17 and CCL22 are produced by Th2-activating DCs [43–45]. It is clear that rapid activation of skin-resident DCs towards both Th1 and Th2 cellular responses is indeed associated with oil-based adjuvants. Late activation of DCs by Al(OH)_3_ could be caused by the ability to create a “vaccine depot” at the site of administration of this particular adjuvant allowing slow antigen release or it could be the result of activation of DCs *via* tissue damage at the site of administration, as this is in fact one of the mechanisms of action of Al(OH)_3_ adjuvants [27,46]. However, the observed decrease of inflammatory response 24 hours after administration of Al(OH)_3_ could be the result of the overreaction that consequently led to the suppression of immune response, which can be triggered by necrotic cell death and release of uric acid at the site of vaccine delivery [47,48]. Based on this observation, we suggest that either the dosage of Al(OH)_3_ for the i.d. vaccination route could be potentially even lower than that used in our experiment or Al(OH)_3_ should be used predominantly for i.m. vaccine formulations.

## Conclusions

Adjuvants are added into vaccine formulations with the aim to enhance immune response. They can also influence balance between production of antigen-specific antibodies and setup of cell-mediated immunity. However, application of different adjuvants can lead to unwanted local reactions. From that, we speculated that the use of various adjuvants can provoke a divergence of responses at the injection site. These can be time-dependent, as demonstrated by opposed activation of DCs by oil-based adjuvants and Al(OH)_3_, and by chemokine as well as cytokine expression during 4 and 24 hours. Both CFA and IFA provoked a prolonged reaction at the injection site of with a tendency to increase it, which led to strong local reaction with time. Meanwhile, Emulsigen and Montanide ISA provided a short local reaction, but still provided an adequate immune response by activation of skin-resident DCs. This leads to both Th1 and Th2 responses.

## Supporting information

S1 Table Individual results of mRNA expression. (XLS)
